# Increased and Imbalanced dNTP Pools Symmetrically Promote Both Leading and Lagging Strand Replication Infidelity

**DOI:** 10.1371/journal.pgen.1004846

**Published:** 2014-12-04

**Authors:** Robert J. Buckland, Danielle L. Watt, Balasubramanyam Chittoor, Anna Karin Nilsson, Thomas A. Kunkel, Andrei Chabes

**Affiliations:** 1 Department of Medical Biochemistry and Biophysics, Umea University, Umea, Sweden; 2 Laboratory for Molecular Infection Medicine Sweden (MIMS), Umea University, Umea, Sweden; 3 Laboratory of Molecular Genetics and Laboratory of Structural Biology, National Institute of Environmental Health Sciences, NIH, DHHS, Research Triangle Park, North Carolina, United States of America; The University of North Carolina at Chapel Hill, United States of America

## Abstract

The fidelity of DNA replication requires an appropriate balance of dNTPs, yet the nascent leading and lagging strands of the nuclear genome are primarily synthesized by replicases that differ in subunit composition, protein partnerships and biochemical properties, including fidelity. These facts pose the question of whether imbalanced dNTP pools differentially influence leading and lagging strand replication fidelity. Here we test this possibility by examining strand-specific replication infidelity driven by a mutation in yeast ribonucleotide reductase, *rnr1-Y285A*, that leads to elevated dTTP and dCTP concentrations. The results for the *CAN1* mutational reporter gene present in opposite orientations in the genome reveal that the rates, and surprisingly even the sequence contexts, of replication errors are remarkably similar for leading and lagging strand synthesis. Moreover, while many mismatches driven by the dNTP pool imbalance are efficiently corrected by mismatch repair, others are repaired less efficiently, especially those in sequence contexts suggesting reduced proofreading due to increased mismatch extension driven by the high dTTP and dCTP concentrations. Thus the two DNA strands of the nuclear genome are at similar risk of mutations resulting from this dNTP pool imbalance, and this risk is not completely suppressed even when both major replication error correction mechanisms are genetically intact.

## Author Summary

The building blocks of DNA, dNTPs, are vital to life, and thus their production is carefully controlled within each cell. Under certain conditions, such as cancer, infection, or drugs, the overall dNTP level or dNTP balance can change. Using yeast genetics we manipulated the dNTP pool balance in unicellular baker's yeast and analysed the effects upon fidelity of DNA replication. We also disrupted mismatch repair, an internal safety system that corrects replication errors and is mutated in many cancers. By sequencing DNA from yeast cells with these alterations we gain insights into the mechanisms of mutation formation that contribute to genome instability. We find that the leading and lagging strand replication fidelity is affected similarly by the dNTP pool imbalance and that the mismatch repair machinery corrects replication errors driven by a dNTP pool imbalance with highly variable efficiencies.

## Introduction

The integrity of an organism's genome is vital to its continued survival, whether unicellular microbe or complex large mammal [1]. Therefore, there are highly conserved mechanisms involved in regulating and protecting genetic material both during and post DNA replication. One of the first safety systems for DNA replication is the stringent control of dNTP synthesis by ribonucleotide reductase (RNR), which maintains concentrations of the individual dNTPs at different levels [1], [2]. RNR catalyses the rate-limiting step in the production of all four dNTPs for the synthesis of nuclear and mitochondrial DNA [3], [4]. In yeast, RNR is a multi-subunit complex comprised of a large subunit, which exist as a homodimer of Rnr1 proteins or a heterodimer of Rnr1/Rnr3 proteins, and a small subunit comprised of Rnr2/Rnr4 proteins. The large subunits contain allosteric specificity sites that modulate enzyme activity and control the balance of the four dNTPs by influencing the specific ribonucleoside diphosphate reduction reaction within the catalytic sites [5]. A highly conserved loop of 13 amino acid residues (Loop 2) connects the allosteric specificity and catalytic sites and is crucial for the correct allosteric regulation of the enzyme [6], [7].

The DNA polymerase selectivity, proofreading and mismatch repair are subsequent safety systems that determine the fidelity of DNA replication. The DNA polymerase selectivity ensures insertion of the correct nucleotide during DNA synthesis. Although the major replicative polymerases alpha (Pol α), delta (Pol δ), and epsilon (Pol ε) are high fidelity enzymes, their accuracy is dependent upon the supply of dNTPs [8]. The second mechanism is proofreading in which errors are removed from primer termini during replication by a 3′–5′ exonuclease activity. Errors that escape proofreading can still be repaired post-replication, through the mismatch repair system (MMR) (reviewed in [9]). The major components of MMR are the homologs of the bacterial MutS proteins, a heterodimer of either Msh2-Msh6 or Msh2-Msh3 that recognise and bind to the mismatch. Msh2-Msh6 is mainly responsible for repairing single base-base mismatches, short insertions and deletions (indels) and small loops, whereas Msh2-Msh3 is involved in larger loop repair. Therefore, Msh2 is essential for MMR [10] and loss of this activity elevates mutation rates [11]. Mutation or loss of Msh2 in humans is associated with microsatellite instability and hereditary nonpolyposis colorectal cancer (HNPCC) [12] and gall bladder cancer [13].

The current model of the eukaryotic replication fork involves DNA polymerase complexes with very different subunit composition, enzymatic properties and fidelity. The leading strand is synthesized primarily by Pol ε, while the lagging strand is synthesized primarily by Pol α and Pol δ [14], [15]. Here we asked whether an imbalanced dNTP pool can force the leading and lagging strand polymerases to produce different errors. It is possible to answer this question by using a gene that is located close to an origin of replication and switching the leading and lagging strand synthesis. We previously created a panel of yeast strains with defined dNTP pool imbalances. The imbalances, in which none of dNTP levels was below normal, did not activate the genome integrity checkpoint and were highly mutagenic despite the availability of functional proofreading and MMR [16]. Utilizing a strain with elevated dTTP and dCTP concentrations and normal dATP and dGTP concentrations, we previously determined the rate and specificity of replication errors generated at the *CAN1* locus [17]. As the *CAN1* reporter gene is located close to a replication origin, by reversing the orientation of *CAN1* and thereby switching the leading and lagging strand synthesis at this locus, we can analyse potential mutational strand bias. To determine the efficiency of DNA mismatch repair in the presence of this dNTP pool imbalance, we also created an *rnr1-Y285A* mutant strain that was MMR deficient. Our data demonstrate that the mutational potential of this dNTP pool imbalance overpowers the intrinsic differences in error specificity of the leading and lagging strand polymerases and reveals that MMR works with highly variable efficiency.

## Results

### dNTP pools of the *rnr1-*Y285A and *msh2*Δ strains

**Figure 1:**
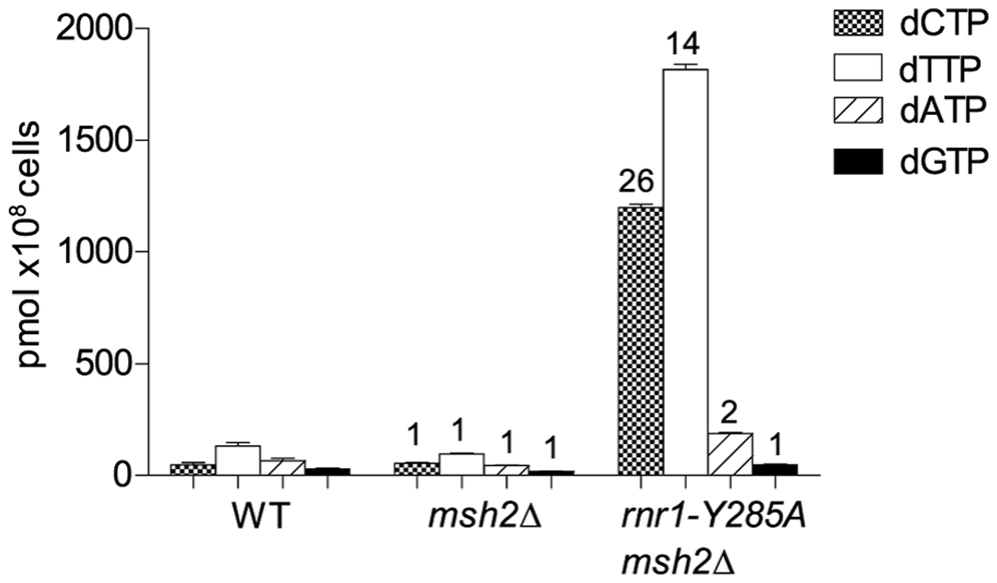
dNTP pools of the strains with the imbalanced dNTP pools. Numbers above columns show the factor increase over wt. Error bars show Standard Error of Mean.

To examine potential differences in mutational specificity between the major replicative polymerases in the presence of the imbalanced dNTP pools, we reversed the orientation of the *CAN1* gene (*CAN1-OR2*). To investigate the effect of this dNTP pool imbalance in the absence of MMR, we deleted *MSH2* in the *rnr1-Y285A CAN*-*OR1* strain. The *msh2*Δ single mutant strain had normal dNTP pools (Fig. 1). The dNTP pools in the double mutant had the same imbalance as in the single *rnr1-Y285A* [17], with approximately 26- and 14-fold higher concentrations of dCTP and dTTP, respectively, compared to wild type (wt) whilst the concentration of dATP was about double and dGTP was normal (Fig. 1).

### *CAN1* spontaneous mutation rates and types

**Figure 2:**
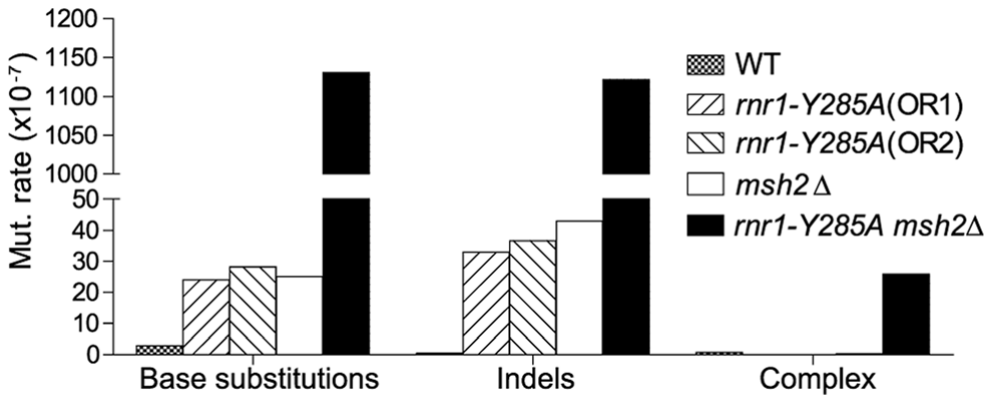
Mutations rates by class.

The spontaneous *CAN1* mutation rate in the *rnr1-Y285A CAN1-OR2* was 13-fold higher than wt (Table 1), which was similar to the *CAN1-OR1* previously published *OR1* [17]. The *msh2*Δ mutant had a 15-fold higher mutation rate compared to wt, however, the *rnr1-Y285A msh2*Δ strain mutation rate was over 500-fold greater than that of wt and over 30-fold either of the relative single mutants. Indels were the major mutation type observed in all four mutant strains whereas it was single base substitutions in wt (Fig. 2). The *rnr1-Y285A*, with *CAN1* in both orientations, and *msh2*Δ strains had an average increase in the indel rate of more than 60-fold the wt strain (0.5×10^7^ for wt versus 33×10^7^ for *OR1* [17], 37× for *OR2*, and 42×10^7^ for *msh2*Δ). However, the double mutant indel rate was increased the most at more than 2000-fold over wt. In addition to single base indels, base substitutions were also significantly increased in the mutants to over 8-fold higher in the single mutants and 350-fold higher in *rnr1-Y285A msh2*Δ compared to wt. Complex mutations, defined as mutations involving insertions or deletions of multiple bases, were much more common in the double mutant, occurring at a rate over 30 times higher than that in wild type.

**Table 1:** *CAN1* mutation rates and observed events.^a^

Strain	wt^a^	*rnr1-Y285A* ^a^	*rnr1-Y285A CAN1* OR2	*msh2*Δ	*rnr1-Y285A msh2*Δ
Mutation rate (x10^-7^)	4.2	57	60	66	2236
95% CI	1.6–4.4	43–103	40–108	53.4–90	1883–3272
*can1* mutants sequenced	93	173	170	164	259
Base substitutions	65	72	80	62	131
Single base indels	11	101	104	106	130
Others	17	0	0	1	3
Total mutations	93	173	184	169	264

Data previously published in [17].

### Mutation hotspots

**Figure 3:**
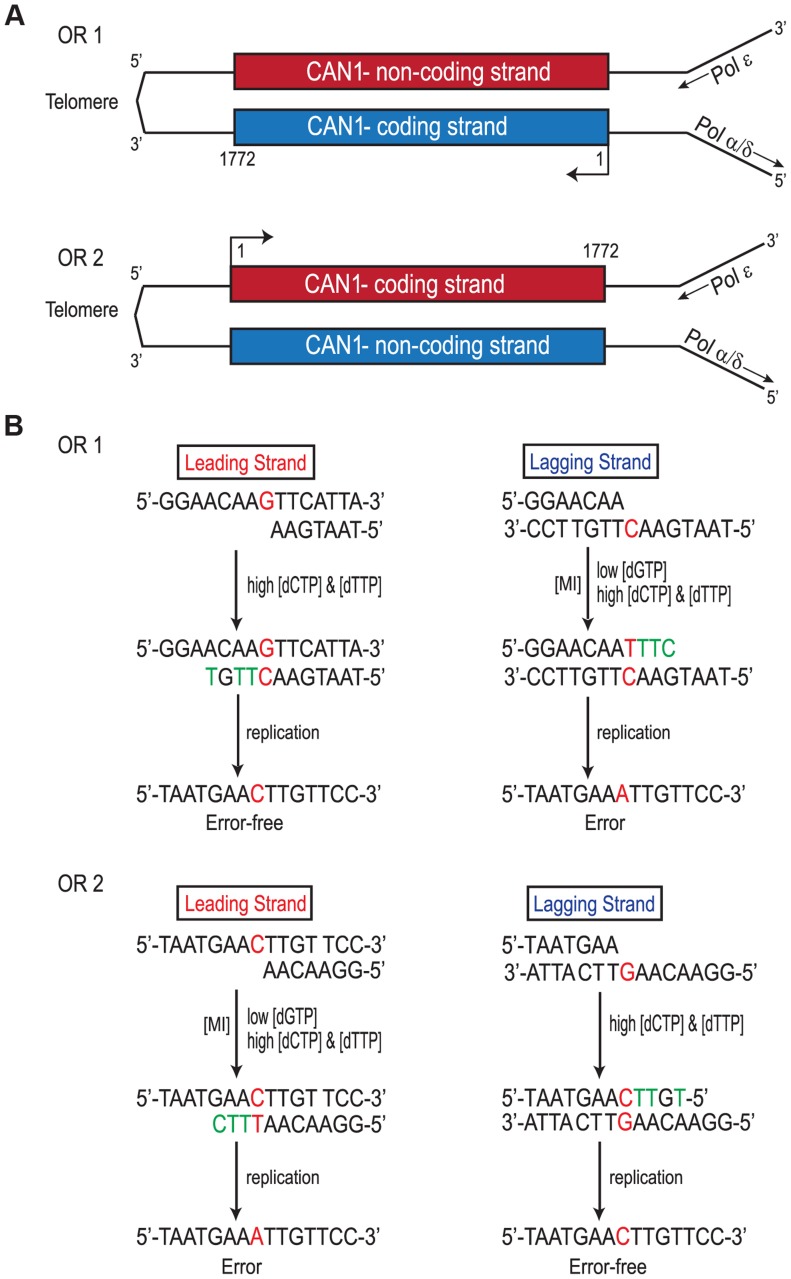
Strand Assignment Model. A. Cartoon representation of </emph>*CAN1* **orientation in** *rnr1-Y285A* **strains.** B. Model showing strand assignment in the two *rnr1-Y285A* mutants (OR1 and OR2) using the hotspot at site 648 bp as an example. MI =  Misinsertion. Red characters represent the mutational event and green characters represent bases where the dNTP is at an excessively high concentration.

Replication of the *CAN1* gene originates from ARS507 and travels through the gene towards the telomere [18]–[20] (Fig. 3A). Therefore, in *rnr1-Y285A CAN1*-*OR1* the leading strand polymerase, presumed to be Pol ε [21], uses the non-coding strand as the template while the coding strand is the template for lagging strand replication primarily by Pol δ or Pol α [14]. In *CAN1*-*OR2*, Pol ε now copies the coding strand and Pol δ/Pol α copy the non-coding strand (see Fig. 3A). An example is given in Fig. 3B, for the single base substitution at 648 bp. During leading strand synthesis in *OR1*, no mistake is made when copying template G due to high concentration of dCTP. However, during lagging strand replication dTTP is inserted opposite template C, as dTTP is at a much higher concentration than the dGTP required for correct pairing. As the succeeding incoming nucleotides are also at an increased concentration, rapid extension then follows stabilizing the C: dT mismatch. In the next round of replication, a C to A mutation arises. When the gene is reversed in *rnr1-Y285A CAN1-OR2*, Pol ε now copies the template C with low fidelity by misinserting dTTP, which ultimately results in a C to A mutation, and Pol δ/Pol α replication is error-free.

**Figure 4:**
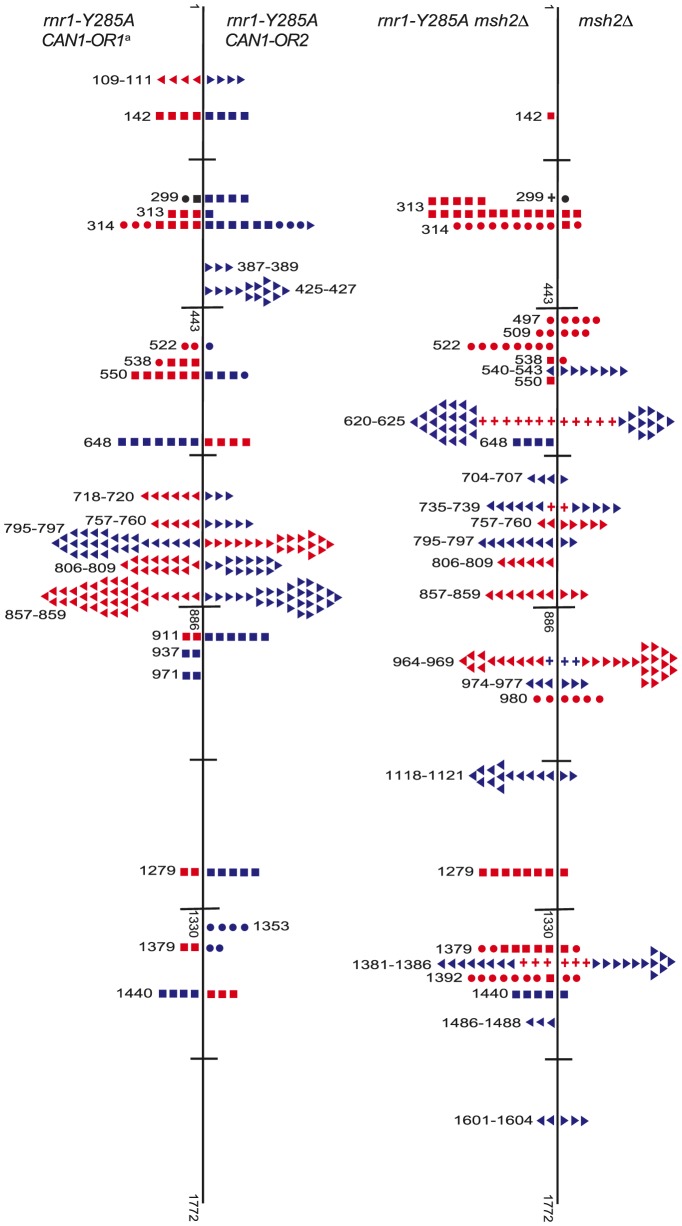
Simplified *CAN1* mutation spectra showing hotpots (where mutation rate is greater than 10-fold that in wt) for *rnr1-Y285A CAN1-OR1* (n = 173), *rnr1-Y285A CAN1-OR2* (n = 170), *rnr1-Y285A msh2*Δ (n = 259), and *msh2*Δ (n = 164) where n =  number of individual colonies sequenced. Symbols indicate the following: plus - additions, triangles - deletions, squares - transversions, circles - transitions, red - occur during leading strand synthesis, blue - occur during lagging strand synthesis, black - mutation cannot be assigned to a strand. ^a^ Reanalysed from the dataset published in [17].

**Figure 5:**
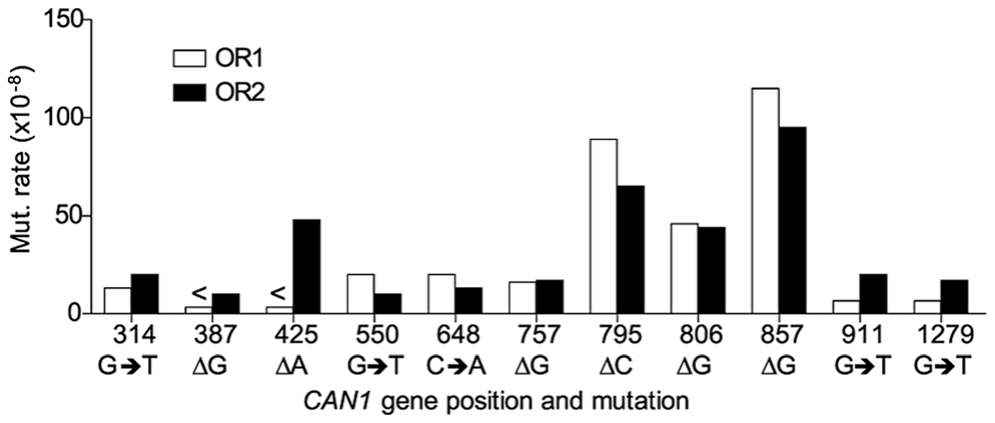
Comparison of *CAN1* mutation rates at hotspots (predominant mutation at site) in *rnr1-Y285A* strains with natural (OR1) and reversed (OR2) orientation of the *CAN1* gene. <Denotes that no events were detected.

Hotspots, mutation sites where the rates were ≥10-fold greater than wt, were assigned to have occurred during leading or lagging strand synthesis by the nature of the mutation observed and dNTP pool imbalance. Simplified mutational spectra illustrating the hotspots in the *CAN1* gene for each strain are shown in Fig. 4 with the full spectra in Figure S1-S3. The hotspot mutation rate was calculated by the equation [(frequency/total no. of samples) x *CAN1* mutation rate]. The majority of hotspots in *rnr1-Y285A CAN1*-*OR1* and *OR2* show no leading – lagging strand bias and have similar mutation rates in both strains (Fig. 5). However, the major hotspot at position 425–427 bp was only seen in *OR2* and had a mutation rate of 48×10^−8^ which was 15-fold higher than in *OR1*.

**Figure 6:**
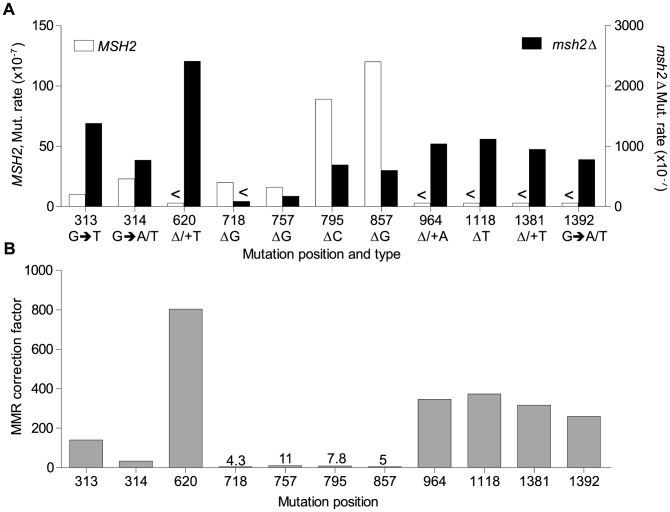
Mismatch repair efficiency. A. Comparison of site mutation rates at major hotspots in *rnr1-Y285A* **with (left axis) and without MMR (right axis).** B. MMR correction factor at these sites in the presence of an *rnr1-Y285A* dNTP pool imbalance. <Denotes no events observed.

The *rnr1-Y285A* and *msh2*Δ strains had several shared major hotspots. Three single base deletions occurred in G: C homopolymeric runs at 757–760 bp, 795–797 bp, and 857–859 bp and two single base substitutions at 313 bp and 1379 bp (Fig. 4 and Table S1). Whilst the double mutant shared these five hotspots with both single mutants, there was a more than 100-fold increase in rates for base substitutions at 313 bp and 13791 bp (Fig. 6A and Table S1). The major hotspots in *rnr1-Y285A msh2Δ* were those seen in *msh2Δ* at 1118–1121 bp, 1392 bp, and especially the dominant deletion hotspots at 620–625 bp, 964–969 bp, and 1381–1386 bp. The site-specific mutation rates in the double mutant ranged from 4- to 800-fold the single mutants.

### MMR efficiency

Analysis of the mutation spectra in the *rnr1-Y285A* and *rnr1-Y285A msh2*Δ strains showed that MMR had different efficiency at distinct mismatches. The ratio of mutation rates in the *msh2Δ* and MMR-proficient strains gave site-specific MMR correction efficiency (Fig. 6B and Table S1). The five hotspots (314, 718, 757, 795 and 857 bp), which include those with the highest mutation rates in the single *rnr1-Y285A* mutant, were the same sites that MMR was the least efficient at repairing errors. The correction factors were less than 30 and only around 10 in four of these sites (i.e., around 10% of errors at these sites will remain uncorrected by MMR). The majority of sites with the highest mutation rates in the double mutant (620, 964, 1118, 1381, and 1392 bp) were those that MMR was best at repairing, namely at T: A mononucleotide repeats. Loss of *MSH2* increased the mutation rate at these sites by 260- to 800-fold.

## Discussion

### Increased dCTP and dTTP drive different polymerases to make similar errors

Despite the inherent differences in complexity of continuous (leading strand) and non-continuous (lagging strand) synthesis, the increased dCTP and dTTP drive the same kind of mutations at identical sequences regardless of the replicative DNA polymerase. Most of the mutations occurred at a G: C base pair in which the cytosine served as the template for synthesis and was often flanked by a 5′-A or a tract of purines as exemplified in Fig. 3. With the concentration of dGTP being the lowest and dCTP and dTTP the highest, the deletion of a G: C base pair in a mononucleotide repeat is stabilized by the rapid incorporation of the next incoming nucleotide (dTTP opposite the template A), as described in detail in our previous report [17]. This dNTP imbalance and sequence context also explains the G: C to T: A base substitutions where dTTP is misinserted opposite template C and mismatch extension proceeds with the rapid incorporation of the pyrimidines opposite the flanking tract of purines. Thus, the mismatch remains at the expense of polymerase proofreading.

However, an exception was found at 425 bp (where there is a hotspot found only when the *CAN1* gene is reversed to OR2). Although similar sequences (AT runs) show no variation in mutation rates between orientations it appears that polymerase δ or α could be making a mistake at this point but not Pol ε. There were also several minor hotspots that suggest polymerase specificity (positions 538, 937, and 971 were unique to OR1 whereas 387 and 1353 were seen only in OR2) which could be indicative of the differences in polymerase efficiency in certain sequence contexts. Whole genome sequencing may give insight into other sites and contexts that affect polymerase specificity and establish the patterns of mutations arising in the presence of this dNTP pool imbalance.

Given that the concentration of dATP was also lower than dCTP and dTTP, T: A to G: C transversions could also be expected in the base substitution hotspots where dCTP is misinserted opposite template T during replication. The nucleotide ratio of dCTP: dATP increased from ∼1∶1 in the wt strain to ∼6∶1 in the *rnr1-Y285A* strains. However, the increase in the nucleotide ratio of dTTP: dGTP was larger, from ∼4∶1 in the wt strain to ∼38∶1 in the *rnr1-Y285A* strains, which may explain the prevalence of the G: C to T: A transversions. Furthermore, the lack of T: A to G: C transversions may be due to the intrinsic difference in the rates at which the two errors are generated. Recent genome-wide studies in *S. cerevisiae* have reported that G: C to T: A transversions were observed at a higher rate than T: A to G: C in strains with normal dNTP pools [22], [23]. The three major replicative polymerases were more prone to generate G: C to T: A transversions but very rarely generated T: A to G: C transversions [23]. In addition, tumours with somatic mutations in the exonuclease domain of Pol ε have a higher prevalence of C to A mutations [24]–[28].

### MMR repairs replication errors driven by a dNTP pool imbalance with highly variable efficiencies

MMR efficiency was dependent upon the site and mismatch generated from the dNTP pool imbalance. The increase in indels in the *msh2*Δ strains was not surprising as MMR is known to be highly active at repairing mistakes at mononucleotide repeats [29], [30]. The indels were almost entirely unique to sequences with ≥3 mononucleotide repeats in the double mutant (99.2%, 127 of 128) compared to 91% in the *msh2Δ* mutant and most frequently occurred in A: T runs. This can be predicted as A-T mononucleotide repeats are often the site of indels in MMR deficient strains [31] and are by far the most common in the *CAN1* gene sequence (Figure S4). Indeed, it appears that the relationship between mutation rate and mononucleotide repeat length is exponential as others have found across the whole yeast genome [22].

The MMR correction factor for all indels in the *rnr1-Y285A* background was 32, which means that on average, 31 of 32 indels are corrected by MMR (compare Fig. 2 rates). Nevertheless, this is ∼3-fold lower than that in the wt RNR background suggesting that some indels driven by this dNTP pool imbalance escape MMR. In addition, there were several major indel hotspots, mainly at G: C base pairs in mononucleotide repeats, with correction factors of 10 compared to the indels at A: T repeats which ranged from 200 to 800. This is a huge variation in the vital post-replication repair machinery that supports the notion of MMR efficiency being dependent on the dNTP pool imbalance, sequence context, and identity of the mismatch.

There are several possibilities as to why MMR is not efficient at these sites in the *rnr1-Y285A* strain. First, there could be a saturation of MMR due to the volume of errors induced by the pool imbalance that are not corrected by proofreading [32]. Consider the hotspots at 795 and 857 bp which dominated the spectra in *rnr1-Y285A*. The correction factor for the wt RNR strain was more than 2- and 5-fold higher than for the *rnr1-Y285A* mutant at the 795 and 857 hotspots, respectively (S1 Table). Therefore, MMR was more accurate at repairing deletions at these sites in the wt RNR strain with normal dNTP pools. Second, MMR itself may require a natural dNTP pool balance in order to correctly repair mistakes. If MMR complexes recognise the mismatches generated but recruit an error-prone or even high fidelity polymerase, the imbalanced dNTP concentration may result in the same mismatch; thus, the mutation is maintained. Finally, some mismatches may not be subjected to MMR if they are damaged or generated outside of DNA replication [33]–[37].

## Materials and Methods

### Yeast strains

The *CAN1* gene was replaced with *URA3* from the pUG72 plasmid [38] (primers “CAN1 Del Ura3” in Materials and Methods S1) in the RNR mutated strain (*rnr1-Y285A*) previously described [16]. PCR amplified *WT CAN1* in reversed orientation (primers “CAN1 orientation” in Materials and Methods S1) was then transformed into the *can1:: URA3* strain to give *rnr1-Y285A CAN1 OR2*. 5-FOA selection allowed the elimination of any *can1:: URA3* cells and the *CAN1* reverse orientation was confirmed by sequencing (“can1ori scr” primers in Materials and Methods S1).

An MMR deficient strain was created by deleting *MSH2* in the AC402 wt (to give *msh2*Δ) using the pAG32 plasmid and transfection technique [39] and the primers msh2_hphMX4, shown in Materials and Methods S1. The deletion was confirmed using primers flanking *MSH2*. This strain was then crossed with *rnr1-Y285A* [16], sporulated and dissected spores selected on Hygromycin and –Trp plates for the double mutant *rnr1-Y285A msh2*Δ.

### Culture conditions and Canavanine resistance assay

All culturing was carried out at 30°C in YPAD (1% yeast extract, 2% bacto-peptone, 20 mg/l adenine, 2% agar for plates) liquid cultures in a shaking incubator at 160rpm. The Canavanine resistance assay was used to calculate mutation rates as previously described [17], [40], [41]. The Can^r^ colonies were picked and the *CAN1* gene amplified and sequenced (MWG Eurofins) using published primers [17] to produce the mutation spectra.

### dNTP pool measurements

dNTP pools were measured in asynchronous cultures as described previously [16] with minor changes as described in [42]. Briefly, cells were harvested by filtration at a density of 0.4×10^7^ to 0.5×10^7^ cells/ml and NTPs and dNTPs were extracted in trichloroacetic acid and MgCl_2_ followed by a Freon-trioctylamine mix. dNTPs were separated using boronate columns (Affigel 601, Biorad) and analysed by HPLC on a LaChrom Elite UV detector (Hitachi) with a Partisphere SAX column (Hichrom, UK).

## Supporting Information

Figure S1 Full *CAN1* mutation spectrum of *rnr1-Y285A CAN1-OR2* strain, showing individual mutations.
(PDF)

Figure S2 Full *CAN1* mutation spectrum of *msh2*Δ strain, showing individual mutations.
(PDF)

Figure S3 Full *CAN1* mutation spectrum of *rnr1-Y285A msh2*Δ strain, showing individual mutations.
(PDF)

Figure S4 Base composition and mutation rates of *CAN1* gene. A. Comparison of hotspot mutation rate and mononucleotide repeat length (all bases) in *msh2*Δ strain. B. Base composition and mononucleotide repeat frequency of wild type *CAN1* gene sequence.
(DOCX)

Table S1 *CAN1* hotspots observed in strains used in this study arranged into speculative classification as follows: (1) are *msh2*Δ hotspots that are enhanced by high dCTP and dTTP levels, (2) are *rnr1-Y285A* hotspots that are enhanced by the loss of MMR, and (3) are hotspots that exist in both single mutants and are enhanced in combination. ^a^ Classification of hotspots observed in the *rnr1-Y285A msh2*Δ double mutant strain. ^b^MMR correction factor for wt and *rnr1-Y285A* strains. ^c^< =  rate calculation based on if one event was observed. ^d^Only one event was observed.
(PDF)

Materials and Methods S1 Primers used in this study.
(DOCX)

## References

[1] Kunz BA, Kohalmi SE, Kunkel TA, Mathews CK, McIntosh EM, et al (1994) International Commission for Protection Against Environmental Mutagens and Carcinogens. Deoxyribonucleoside triphosphate levels: a critical factor in the maintenance of genetic stability. Mutation research 318: 1–64. 7519315 10.1016/0165-1110(94)90006-x

[2] Mathews CK (2014) Deoxyribonucleotides as genetic and metabolic regulators. FASEB J. E-pub ahead of print. 10.1096/fj.14-251249 PMC5395721 24928192

[3] Reichard P (1988) Interactions between deoxyribonucleotide and DNA synthesis. Annual review of biochemistry 57: 349–374. 10.1146/annurev.bi.57.070188.002025 3052277

[4] Thelander L (2007) Ribonucleotide reductase and mitochondrial DNA synthesis. Nature genetics 39: 703–704. 17534360 10.1038/ng0607-703

[5] Thelander L, Reichard P (1979) Reduction of ribonucleotides. Annual review of biochemistry 48: 133–158. 10.1146/annurev.bi.48.070179.001025 382982

[6] Hofer A, Crona M, Logan DT, Sjoberg BM (2012) DNA building blocks: keeping control of manufacture. Critical reviews in biochemistry and molecular biology 47: 50–63. 22050358 10.3109/10409238.2011.630372 PMC3267527

[7] Xu H, Faber C, Uchiki T, Fairman JW, Racca J, et al (2006) Structures of eukaryotic ribonucleotide reductase I provide insights into dNTP regulation. Proceedings of the National Academy of Sciences of the United States of America 103: 4022–4027. 16537479 10.1073/pnas.0600443103 PMC1389704

[8] Yao NY, Schroeder JW, Yurieva O, Simmons LA, O'Donnell ME (2013) Cost of rNTP/dNTP pool imbalance at the replication fork. Proceedings of the National Academy of Sciences 110: 12942–12947. 10.1073/pnas.1309506110 PMC3740908 23882084

[9] Kunkel TA, Erie DA (2005) DNA mismatch repair. Annual review of biochemistry 74: 681–710. 10.1146/annurev.biochem.74.082803.133243 15952900

[10] Harfe BD, Jinks-Robertson S (2000) DNA mismatch repair and genetic instability. Annual review of genetics 34: 359–399. 10.1146/annurev.genet.34.1.359 11092832

[11] Johnson RE, Kovvali GK, Prakash L, Prakash S (1996) Requirement of the Yeast MSH3 and MSH6 Genes for MSH2-dependent Genomic Stability. Journal of Biological Chemistry 271: 7285–7288. 8631743 10.1074/jbc.271.13.7285

[12] Peltomaki P, Vasen HF (1997) Mutations predisposing to hereditary nonpolyposis colorectal cancer: Database and results of a collaborative study. The International Collaborative Group on Hereditary Nonpolyposis Colorectal Cancer. Gastroenterology 113: 1146–1158. 9322509 10.1053/gast.1997.v113.pm9322509

[13] Srivastava K, Srivastava A, Mittal B (2010) Polymorphisms in ERCC2, MSH2, and OGG1 DNA repair genes and gallbladder cancer risk in a population of Northern India. Cancer 116: 3160–3169. 20564624 10.1002/cncr.25063

[14] Nick McElhinny SA, Gordenin DA, Stith CM, Burgers PM, Kunkel TA (2008) Division of labor at the eukaryotic replication fork. Molecular Cell 30: 137–144. 18439893 10.1016/j.molcel.2008.02.022 PMC2654179

[15] Burgers PMJ (2009) Polymerase Dynamics at the Eukaryotic DNA Replication Fork. Journal of Biological Chemistry 284: 4041–4045. 18835809 10.1074/jbc.R800062200 PMC2640984

[16] Kumar D, Viberg J, Nilsson AK, Chabes A (2010) Highly mutagenic and severely imbalanced dNTP pools can escape detection by the S-phase checkpoint. Nucleic Acids Research 38: 3975–3983. 20215435 10.1093/nar/gkq128 PMC2896522

[17] Kumar D, Abdulovic AL, Viberg J, Nilsson AK, Kunkel TA, et al (2011) Mechanisms of mutagenesis in vivo due to imbalanced dNTP pools. Nucleic Acids Research 39: 1360–1371. 20961955 10.1093/nar/gkq829 PMC3045583

[18] Raghuraman MK, Winzeler EA, Collingwood D, Hunt S, Wodicka L, et al (2001) Replication dynamics of the yeast genome. Science 294: 115–121. 11588253 10.1126/science.294.5540.115

[19] Yabuki N, Terashima H, Kitada K (2002) Mapping of early firing origins on a replication profile of budding yeast. Genes to cells: devoted to molecular & cellular mechanisms 7: 781–789. 12167157 10.1046/j.1365-2443.2002.00559.x

[20] Kim H-M, Narayanan V, Mieczkowski PA, Petes TD, Krasilnikova MM, et al (2008) Chromosome fragility at GAA tracts in yeast depends on repeat orientation and requires mismatch repair. EMBO J 27: 2896–2906. 18833189 10.1038/emboj.2008.205 PMC2580784

[21] Pursell ZF, Isoz I, Lundstrom EB, Johansson E, Kunkel TA (2007) Yeast DNA polymerase epsilon participates in leading-strand DNA replication. Science 317: 127–130. 17615360 10.1126/science.1144067 PMC2233713

[22] Serero A, Jubin C, Loeillet S, Legoix-Né P, Nicolas AG (2014) Mutational landscape of yeast mutator strains. Proceedings of the National Academy of Sciences 111: 1897–1902 doi:10.1073/pnas.1314423111 10.1073/pnas.1314423111 PMC3918763 24449905

[23] Lujan SA, Clausen AR, Clark AB, MacAlpine HK, MacAlpine DM, et al. (2014) Heterogeneous polymerase fidelity and mismatch repair bias genome variation and composition. Genome Res. doi:10.1101/gr.178335.114 10.1101/gr.178335.114 PMC4216917 25217194

[24] Kane DP, Shcherbakova PV (2014) A common cancer-associated DNA polymerase epsilon mutation causes an exceptionally strong mutator phenotype, indicating fidelity defects distinct from loss of proofreading. Cancer Research 74: 1895–1901. 24525744 10.1158/0008-5472.CAN-13-2892 PMC4310866

[25] Shinbrot E, Henninger EE, Weinhold N, Covington KR, Goksenin AY, et al. (2014) Exonuclease mutations In DNA Polymerase Epsilon reveal replication strand specific mutation patterns and human origins of replication. Genome Research. doi:10.1101/gr.174789.114 10.1101/gr.174789.114 PMC4216916 25228659

[26] Alexandrov LB, Nik-Zainal S, Wedge DC, Aparicio SA, Behjati S, et al (2013) Signatures of mutational processes in human cancer. Nature 500: 415–421. 23945592 10.1038/nature12477 PMC3776390

[27] Muzny DM, Bainbridge MN, Chang K, Dinh HH, Drummond JA, et al (2012) Comprehensive molecular characterization of human colon and rectal cancer. Nature 487: 330–337. 22810696 10.1038/nature11252 PMC3401966

[28] Getz G, Gabriel SB, Cibulskis K, Lander E, Sivachenko A, et al (2013) Integrated genomic characterization of endometrial carcinoma. Nature 497: 67–73. 23636398 10.1038/nature12113 PMC3704730

[29] Lujan SA, Williams JS, Pursell ZF, Abdulovic-Cui AA, Clark AB, et al (2012) Mismatch Repair Balances Leading and Lagging Strand DNA Replication Fidelity. PLoS Genet 8: e1003016. 23071460 10.1371/journal.pgen.1003016 PMC3469411

[30] Gragg H, Harfe BD, Jinks-Robertson S (2002) Base composition of mononucleotide runs affects DNA polymerase slippage and removal of frameshift intermediates by mismatch repair in Saccharomyces cerevisiae. Mol Cell Biol 22: 8756–8762. 12446792 10.1128/MCB.22.24.8756-8762.2002 PMC139878

[31] Lehner K, Mudrak SV, Minesinger BK, Jinks-Robertson S (2012) Frameshift mutagenesis: the roles of primer-template misalignment and the nonhomologous end-joining pathway in Saccharomyces cerevisiae. Genetics 190: 501–510. 22095081 10.1534/genetics.111.134890 PMC3276632

[32] Schaaper RM, Radman M (1989) The extreme mutator effect of Escherichia coli mutD5 results from saturation of mismatch repair by excessive DNA replication errors. The EMBO journal 8: 3511–3516. 2555167 10.1002/j.1460-2075.1989.tb08516.x PMC401508

[33] Kramer B, Kramer W, Fritz HJ (1984) Different base/base mismatches are corrected with different efficiencies by the methyl-directed DNA mismatch-repair system of E. coli. Cell 38: 879–887. 6386179 10.1016/0092-8674(84)90283-6

[34] Dohet C, Wagner R, Radman M (1985) Repair of defined single base-pair mismatches in Escherichia coli. Proceedings of the National Academy of Sciences of the United States of America 82: 503–505. 16593539 10.1073/pnas.82.2.503 PMC397067

[35] Schaaper RM, Dunn RL (1991) Spontaneous mutation in the Escherichia coli lacI gene. Genetics 129: 317–326. 1660424 10.1093/genetics/129.2.317 PMC1204625

[36] Schaaper RM (1993) Base selection, proofreading, and mismatch repair during DNA replication in Escherichia coli. The Journal of biological chemistry 268: 23762–23765. 8226906

[37] Nick McElhinny SA, Kissling GE, Kunkel TA (2010) Differential correction of lagging-strand replication errors made by DNA polymerases {alpha} and {delta}. Proceedings of the National Academy of Sciences of the United States of America 107: 21070–21075. 21041657 10.1073/pnas.1013048107 PMC3000245

[38] Gueldener U, Heinisch J, Koehler GJ, Voss D, Hegemann JH (2002) A second set of loxP marker cassettes for Cre-mediated multiple gene knockouts in budding yeast. Nucleic Acids Research 30: e23. 11884642 10.1093/nar/30.6.e23 PMC101367

[39] Goldstein AL, McCusker JH (1999) Three new dominant drug resistance cassettes for gene disruption in Saccharomyces cerevisiae. Yeast 15: 1541–1553. 10514571 10.1002/(SICI)1097-0061(199910)15:14<1541::AID-YEA476>3.0.CO;2-K

[40] Drake JW (1991) A constant rate of spontaneous mutation in DNA-based microbes. Proceedings of the National Academy of Sciences of the United States of America 88: 7160–7164. 1831267 10.1073/pnas.88.16.7160 PMC52253

[41] Lea DE, Coulson CA (1949) The distribution of the numbers of mutants in bacterial populations. Journal of genetics 49: 264–285. 24536673 10.1007/BF02986080

[42] Hoch NC, Chen ES, Buckland R, Wang SC, Fazio A, et al (2013) Molecular basis of the essential s phase function of the rad53 checkpoint kinase. Molecular and cellular biology 33: 3202–3213. 23754745 10.1128/MCB.00474-13 PMC3753913

