# Supplementary material for: Increased and Imbalanced dNTP Pools Symmetrically Promote Both Leading and Lagging Strand Replication Infidelity
Source: PLoS Genet. 2014 Dec 4;10(12):e1004846. doi: 10.1371/journal.pgen.1004846 (PMC4256292; doi:10.1371/journal.pgen.1004846)
Supplement: Figure S3 — Full CAN1 mutation spectrum of rnr1-Y285A msh2Δ strain, showing individual mutations. (PDF) [file pgen.1004846.s003.pdf]

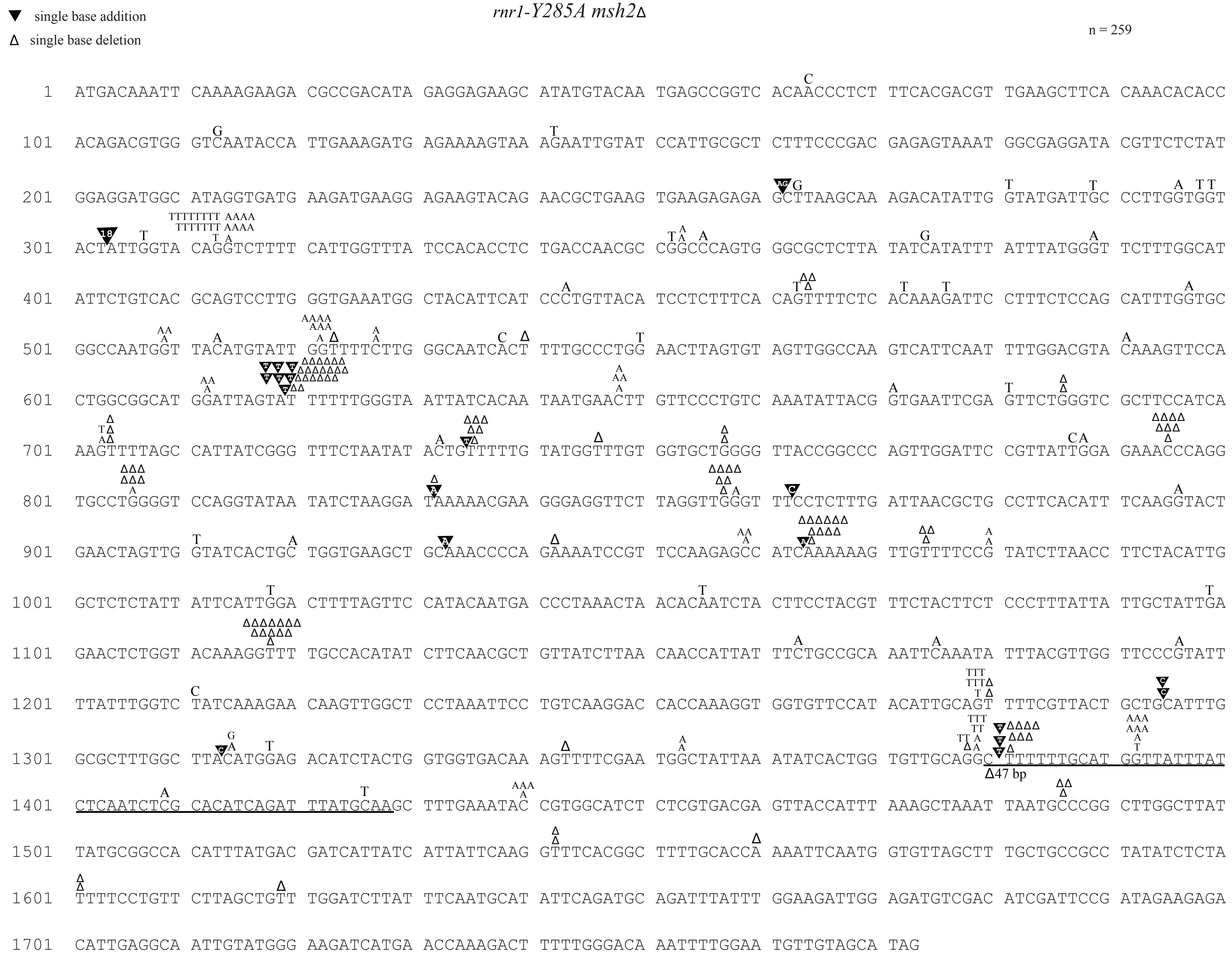

rnr1-Y285A msh2Δ
▼ single base addition
Δ single base deletion
n = 259
1 ATGACAAATT CAAAAGAAGA CGCCGACATA GAGGAGAAGC ATATGTACAA TGAGCCGGTC ACAACCCTCT TTCACGACGT TGAAGCTTCA CAAACACACC
101 ACAGACGTGG GTCAATACCA TTGAAAGATG AGAAAAGTAA AGAATTGTAT CCATTGCGCT CTTTCCCGAC GAGAGTAAAT GGCGAGGATA CGTTCTCTAT
201 GGAGGATGGC ATAGGTGATG AAGATGAAGG AGAAGTACAG AACGCTGAAG TGAAGAGAGA GCTTAAGCAA AGACATATTG GTATGATTGC CCTTGGTGGT
301 ACTATTGGTA CAGGTCTTTT CATTGGTTTA TCCACACCTC TGACCAACGC CGGCCCAGTG GGCGCTCTTA TATCATATTT ATTTATGGGT TCTTTGGCAT
401 ATTCTGTCAC GCAGTCCTTG GGTGAAATGG CTACATTCAT CCCTGTTACA TCCTCTTTCA CAGTTTTCTC ACAAAGATTC CTTTCTCCAG CATTTGGTGC
501 GGCCAATGGT TACATGTATT GGTTTTCTTG GGCAATCACT TTTGCCCTGG AACTTAGTGT AGTTGGCCAA GTCATTCAAT TTTGGACGTA CAAAGTTCCA
601 CTGGCGGCAT GGATTAGTAT TTTTTGGGTA ATTATCACAA TAATGAACTT GTTCCCTGTC AAATATTACG GTGAATTCGA GTTCTGGGTC GCTTCCATCA
701 AAGTTTTAGC CATTATCGGG TTTCTAATAT ACTGTTTTTG TATGGTTTGT GGTGCTGGGG TTACCGGCCC AGTTGGATTC CGTTATTGGA GAAACCCAGG
801 TGCCTGGGGT CCAGGTATAA TATCTAAGGA TAAAAACGAA GGGAGGTTCT TAGGTTGGGT TTCCTCTTTG ATTAACGCTG CCTTCACATT TCAAGGTACT
901 GAACTAGTTG GTATCACTGC TGGTGAAGCT GCAAACCCCA GAAAATCCGT TCCAAGAGCC ATCAAAAAAG TTGTTTTCCG TATCTTAACC TTCTACATTG
1001 GCTCTCTATT ATTCATTGGA CTTTTAGTTC CATACAATGA CCCTAAACTA ACACAATCTA CTTCCTACGT TTCTACTTCT CCCTTTATTA TTGCTATTGA
1101 GAACTCTGGT ACAAAGGTTT TGCCACATAT CTTCAACGCT GTTATCTTAA CAACCATTAT TTCTGCCGCA AATTCAAATA TTTACGTTGG TTCCCGTATT
1201 TTATTTGGTC TATCAAAGAA CAAGTTGGCT CCTAAATTCC TGTCAAGGAC CACCAAAGGT GGTGTTCCAT ACATTGCAGT TTTCGTTACT GCTGCATTTG
1301 GCGCTTTGGC TTACATGGAG ACATCTACTG GTGGTGACAA AGTTTTCGAA TGGCTATTAA ATATCACTGG TGTTGCAGGC TTTTTTGCAT GGTTATTTAT
Δ47 bp
1401 CTCAATCTCG CACATCAGAT TTATGCAAGC TTTGAAATAC CGTGGCATCT CTCGTGACGA GTTACCATTT AAAGCTAAAT TAATGCCCGG CTTGGCTTAT
1501 TATGCGGCCA CATTTATGAC GATCATTATC ATTATTCAAG GTTTCACGGC TTTTGCACCA AAATTCAATG GTGTTAGCTT TGCTGCCGCC TATATCTCTA
1601 TTTTCCTGTT CTTAGCTGTT TGGATCTTAT TTCAATGCAT ATTCAGATGC AGATTTATTT GGAAGATTGG AGATGTCGAC ATCGATTCCG ATAGAAGAGA
1701 CATTGAGGCA ATTGTATGGG AAGATCATGA ACCAAAGACT TTTTGGGACA AATTTTGGAA TGTTGTAGCA TAG
