# Supplementary material for: Increased and Imbalanced dNTP Pools Symmetrically Promote Both Leading and Lagging Strand Replication Infidelity
Source: PLoS Genet. 2014 Dec 4;10(12):e1004846. doi: 10.1371/journal.pgen.1004846 (PMC4256292; doi:10.1371/journal.pgen.1004846)
Supplement: Figure S4 — Base composition and mutation rates of CAN1 gene. A. Comparison of hotspot mutation rate and mononucleotide repeat length (all bases) in msh2Δ strain. B. Base composition and mononucleotide repeat frequency of wild type CAN1 gene sequence. (DOCX) [file pgen.1004846.s004.docx]

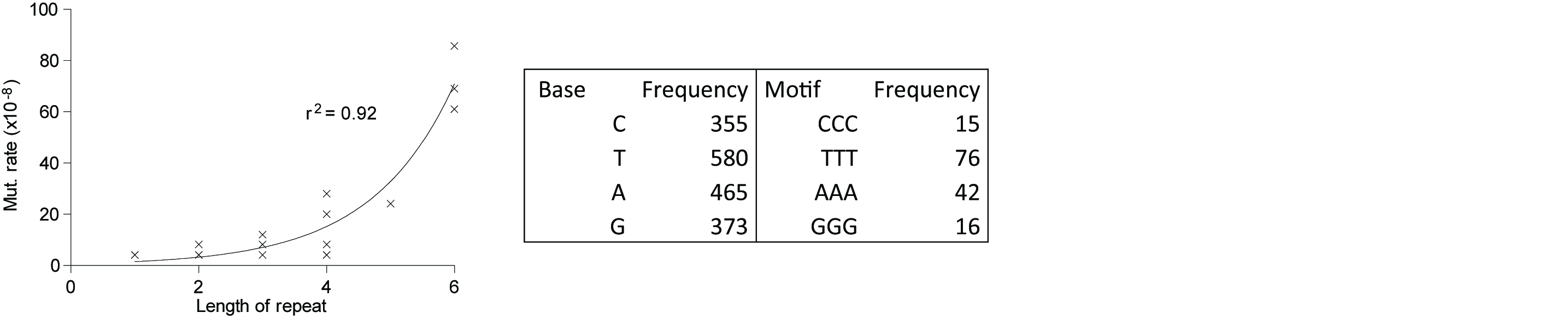


Suppl. Figure 4. Base composition and mutation rates of *CAN1* gene. A. Comparison of hotspot mutation rate and mononucleotide repeat length (all bases) in *msh2*Δ strain. B. Base composition and mononucleotide repeat frequency of wild type *CAN1* gene sequence.
