# Supplementary material for: Increased and Imbalanced dNTP Pools Symmetrically Promote Both Leading and Lagging Strand Replication Infidelity
Source: PLoS Genet. 2014 Dec 4;10(12):e1004846. doi: 10.1371/journal.pgen.1004846 (PMC4256292; doi:10.1371/journal.pgen.1004846)
Supplement: Table S1 — CAN1 hotspots observed in strains used in this study arranged into speculative classification as follows: (1) are msh2Δ hotspots that are enhanced by high dCTP and dTTP levels, (2) are rnr1-Y285A hotspots that are enhanced by the loss of MMR, and (3) are hotspots that exist in both single mutants and are enhanced in combination. a Classification of hotspots observed in the rnr1-Y285A msh2Δ double mutant strain. bMMR correction factor for wt and rnr1-Y285A strains. c< = rate calculation based on if one event was observed. dOnly one event was observed. (PDF) [file pgen.1004846.s005.pdf]

Strain	WT	*msh2*Δ	*rnr1-Y285A*	*rnr1-Y285A msh2*Δ
Rates	4.2 x10^{-7}	67 x10^{-7}	57 x10^{-7}	2236 x10^{-7}
# Individuals	94	164	173	259

Classification[a]	Hotspot	Mutation	**Rate x 10-8**				MMR cf[b]	
			WT	Δ*msh2*	*rnr1-Y285A*	*rnr1-Y285A msh2*Δ	WT	*rnr1-Y285A*
1	540-543	ΔT	< 0.45[c]	28	< 3.0	86[d]	>62	>29
	620-625	ΔT	0,45	49	< 3.0	1810	110	>600
		+T	0,90	20	< 3.0	600	22	>200
		Total	1,3	69	< 3.0	2410	53	>800
	735-739	ΔT	< 0.45	20	3,0	520	>44	170
		+T	< 0.45	4,1	< 3.0	86[d]	>9	>29
		Total	< 0.45	24	3,0	600	>53	200
	964-969	ΔA	< 0.45	77	< 3.0	950	>170	>320
		+A	< 0.45	8,2	< 3.0	86[d]	>18	>29
		Total	< 0.45	86	< 3.0	1040	>190	>350
	1118-1121	ΔT	< 0.45	8,2	< 3.0	1120	>18	>370
	1381-1386	ΔT	< 0.45	49	< 3.0	690	>150	>230
		+T	< 0.45	12	< 3.0	260	>27	>87
		Total	< 0.45	61	< 3.0	950	>140	>320
	1392	G → A	< 0.45	8,2	< 3.0	690	>18	>230
		G → T	< 0.45	< 4.1	< 3.0	86[d]	>9	>29
		Total	< 0.45	8.2	< 3.0	780	>18	>260
2	109-111	ΔG	< 0.45	< 4.1	13	< 86	>9	>7
	142	G → T	< 0.45	< 4.1	13	86[d]	>9	7
	522	G → A	< 0.45	< 4.1	6,6	690	>9	100
	648	C → A	< 0.45	< 4.1	20	340	>9	17
	718-720	ΔG	< 0.45	< 4.1	20	< 86	>9	>4
	806-809	ΔG	< 0.45	< 4.1	46	520	>9	11
	1279	G → T	0,45	4,1	6,0	600	9	100
	1440	C → A	< 0.45	< 4.1	13	340	>9	26
2 or 3<br>Note *msh2* Δ not >10x wt	314	G → A	0,9	4,1	9,9	770	5	78
		G → T	< 0.45	4,1	13	< 86	>9	>29
		Total	0,9	8,2	23	770	9	33
3	313	G → T	< 0.45	8,2	9,9	1380	>18	140
	757-760	ΔG	< 0.45	20	16	170	>44	11
	795-797	ΔC	< 0.45	8,2	89	690	>18	8
	857-859	ΔG	< 0.45	12	120	600	>27	5
	1379	G → T	< 0.45	4,1	6,0	430	>9	72
		G → A	< 0.45	4,1	< 3.0	170	>9	>57
		Total	< 0.45	8,2	6,0	600	>18	100
