# Supplementary material for: Increased and Imbalanced dNTP Pools Symmetrically Promote Both Leading and Lagging Strand Replication Infidelity
Source: PLoS Genet. 2014 Dec 4;10(12):e1004846. doi: 10.1371/journal.pgen.1004846 (PMC4256292; doi:10.1371/journal.pgen.1004846)
Supplement: Materials and Methods S1 — Primers used in this study. (DOCX) [file pgen.1004846.s006.docx]

Primers used in this study.

Primers used for the creation of *MSH2* deletion strain (used pAG32 plasmid);

(msh2_hphMX4_forward); CTGTAAAAAATCTCTTTATCTGCTGACCTAACATCAAAATCCTCAGATTAAAAGTCACATACGATTTAGGTGACAC

(msh2_hphMX4_reverse); ATCTATATATTATCTATCGATTCTCACTTAAGATGTCGTTGTAATATTAATTTGTAATACGACTCACTATAGGGAG

Primers for checking the deletion of *MSH2*;

msh2_forward; AATCCAATCAGAACTCCAGCA

msh2_reverse; CGGAGATACTCTTTCCAGTGG

These give a product of 3468bp in *WT* *MSH2* and 2660bp in *msh2*∆::Hyg

To delete the *CAN1* gene (33466-31694 Chr. V);

| CAN1 Del Ura3 For | 5´-TACAGGCAACAAGTGATAGAGGGCCCATTATGAATACGCA  CCTCTATGTATTTCCCAATACAACAGATCACGTGATC-3´ |
| --- | --- |
| CAN1 Del Ura3 Rev | 5´-GCGCTTACTACTTTTTGGCGTTTTTGCCTATTTCACTATT  TACATATCGTGAAAAGTTTTATTTAGGTTCTATCGAGG-3´ |

Primer binding sites in *CAN1* (in black); 34245-34299 and 31303-31357, respectively. Sites in pUG72 plasmid (in red); 4621-4642 and 3227-3249, respectively.

To reverse the orientation of the *CAN1* gene;

| CAN1 orientation For | 5´-GCGCTTACTACTTTTTGGCGTTTTTGCCTATTTCACTATT  TACATATCGTGAAAAGAGATACGATTACTCCAGTTC-3´ |
| --- | --- |
| CAN1 orientation Rev | 5´-TACAGGCAACAAGTGATAGAGGGCCCATTATGAATACGCA  CCTCTATGTATTTCCTGACATTTGGTTCTAGGTTCGG-3´ |

Primer binding sites in *CAN1* (in black); 34245-34299 and 31303-31357, respectively, for homology in strain created above. Sites in *CAN1* in red to reverse gene; 34224-34244 and 31358-31379, respectively.

To screen and sequence the *CAN1* orientation;

| can1ori scr up for | 5´-CTGACCATTCCCTTTAGTAGAGA-3´ |
| --- | --- |
| can1ori scr up rev | 5´-TCACGTCACCCGAACCT-3´ |
| can1ori scr down for | 5´-ATCAAGGCTAATAAGGGACAAG-3´ |
| can1ori scr down rev | 5´-CTAACTCAGACATTATCGGAACAT-3´ |
